# Omega-3 Fatty Acids and Coronary Artery Disease: More Questions Than Answers

**DOI:** 10.3390/jcm10112495

**Published:** 2021-06-04

**Authors:** Marijana Tadic, Carla Sala, Guido Grassi, Giuseppe Mancia, Stefano Taddei, Wolfgang Rottbauer, Cesare Cuspidi

**Affiliations:** 1 Clinic for Internal Medicine II, Cardiology Department, University Clinic of Ulm, Albert-Einstein Allee 23, 89081 Ulm, Germany; wolfgang.rottbauer@uniklinik-ulm.de; 2 Department of Clinical Sciences and Community Health, University of Milano and Fondazione Ospedale Maggiore IRCCS Policlinico di, 20126 Milan, Italy; carla.sala@unimi.it; 3 Clinica Medica, Cardiology Department, University of Milan-Bicocca, 20126 Milan, Italy; guido.grassi@unimib.it; 4 Department of Medicine and Surgery, University of Milano-Bicocca, Milan and Policlinico di Monza, 28100 Monza, Italy; giuseppe.mancia@unimib.it (G.M.); cesare.cuspidi@unimib.it (C.C.); 5 Department of Clinical and Experimental Medicine, University of Pisa, 56100 Pisa, Italy; stefano.taddei@med.unipi.it; 6 Department of Medicine and Surgery, Milano Istituto Auxologico Italiano, University of Milano-Bicocca, 20126 Milan, Italy

**Keywords:** omega-3 fatty acids, cardiovascular outcome, hypertriglyceridemia

## Abstract

Studies show that patients with elevated triglycerides and well-controlled LDL levels under statin therapy still have a significant residual risk of cardiovascular (CV) events. Despite many attempts to reduce triglycerides with different hypolipidemic drugs, no therapeutic option has given satisfactory results so far. The initial enthusiasm that omega-3 fatty acids can effectively reduce triglycerides and CV risk was replaced with skepticism when the first large clinical trials failed to show any benefit in primary or secondary prevention. However, the latest studies succeeded in showing a positive effect of omega-3 fatty acids on CV outcome in patients with hypertriglyceridemia. The largest benefit was reported in secondary but not primary prevention. Interestingly, the reduction in triglycerides in some of these studies was disproportionately low to the relatively high CV risk reduction, which could indicate some other effects of omega-3 fatty acids that go well beyond hypotriglyceridemic action. This includes blood pressure reduction, antithrombotic effect, improvement of inflammatory status, endothelial function, and insulin resistance. Investigations also reported a significant and positive influence of omega-3 fatty acids on the composition and stabilization of coronary atherosclerotic plaques in patients with and without previous CV events. In addition to insufficiently known mechanisms of action and conflicting results about the effectiveness of omega-3 fatty acids, the safety problems, which include increased prevalence of atrial fibrillation and hemorrhage, were also reported. The aim of this clinical review was to summarize the current knowledge regarding the use of omega-3 fatty acids in CV patients, particularly those with coronary artery disease, and to present an overview of key clinical trial data.

## 1. Introduction

The low-density lipoprotein (LDL) level has been established as an important cardiovascular (CV) risk factor with linear association between it and CV morbidity and mortality, which is the reason for the continuous reduction in recommended LDL values in patients with different levels of CV risk [1]. The role of triglycerides in the CV continuum is less clear. Studies show the association between triglyceride levels and CV outcome, but it is difficult to differentiate the effect of triglycerides independently of LDL and more importantly from other comorbidities (obesity, metabolic syndrome, and diabetes) that are associated with an elevation of triglycerides [2].

The influence of triglycerides on CV outcome still needs to be determined due to conflicting results coming from large studies. On one side, results show a negative association between triglyceride levels and coronary heart disease [3]. On the other side, studies reveal an inverse relationship between low triglyceride levels and risk of all-cause mortality and cardiovascular mortality in patients with confirmed coronary artery disease (CAD) or acute myocardial infarction [4,5]. These provocative conclusions initiated an ongoing debate about the effect of triglycerides in CAD patients.

The potential impact of triglyceride-lowering medications, and particularly omega-3 fatty acids, on CAD is even more controversial, and discussion about their usage in these patients is more active than ever after the latest findings from trials were recently published [6,7,8]. A meta-analysis that investigated the effect of different pharmacological agents in the treatment of 56,471 patients with hypertriglyceridemia showed that simvastatin had a clear advantage in reducing the risk of major adverse cardiovascular events (MACE) [9]. Eicosapentaenoic acid was considered an effective triglycerides-lowering medication [9]. Gemfibrozil was the most effective among fibrates, even though bezafibrate also significantly reduced the risk of MACE in populations with increased triglyceride levels [9]. 

The largest meta-analysis that investigated the effect of omega-3 fatty acids in primary and secondary prevention of CVD showed moderate- and low-certainty evidence about the relationship between the use of omega-3 fatty acids and a slightly reduced risk of coronary heart disease mortality and events and reduced serum triglycerides [10]. 

The aim of this review was to summarize the current knowledge regarding the use of omega-3 fatty acids in CAD patients and to provide a practical approach for clinicians treating these patients.

## 2. The Influence of Triglycerides on Atherosclerosis

Triglycerides are not directly involved in the process of atherosclerosis, but triglyceride-rich lipoproteins, such as very low-density lipoproteins (VLDL), chylomicrons, and their fragments, seem to be associated with atherogenesis independently of LDL. Similar to oxidized LDL, these triglyceride-enriched lipids are accumulated by macrophages in the subendothelial layer and form foam cells that promote fatty streak formation, the precursor of atherosclerotic plaque [11,12]. Hypertriglyceridemia is related to increased VLDL production and delayed VLDL clearance from circulation. Increased liver secretion of VLDL containing apo C-III is frequently found in hypertriglyceridemia and may sometimes be associated with insulin resistance. 

Moreover, delayed clearance of all apo-B lipoproteins, including VLDL, has been ascribed to various mechanisms such as apo C-III-mediated inhibition of apo E and apo B-100 binding to hepatic receptors and proteoglycan, reduced activity of lipoprotein lipase, hepatic triglyceride lipase, or novel receptors such as GPI-anchored HDL-binding protein 1 or proteoglycan [13,14,15]. The liver uses LDL receptors to take up chylomicrons, which are not considered atherogenic due to their large size and inability to enter into the arterial intima. However, the chylomicron fragments are sufficiently small to pass through the endothelial cell barrier to the arterial intima, and therefore are considered atherogenic [14,15]. 

Experimental studies have described mechanisms by which triglyceride-rich lipoproteins and their fragments produce many proatherogenic responses involving the attachment of monocytes through the action of endothelial- and macrophage-derived inflammatory proteins such as tumor necrosis factor-α (TNF-α), interleukin (IL)-1β, intercellular adhesion molecule-1, and matrix metalloproteinase-3 [16]. Triglycerides-rich lipoproteins may suppress the atheroprotective and anti-inflammatory effects of HDL by blocking sterol efflux from monocytes and macrophages [17].

A major possibility for regulation of CVD progression by omega-3 fatty acids is the production of a wide variety of eicosanoid-like mediators. Potently anti-inflammatory effects are induced by P450-derived epoxides and lipoxygenase-derived pro-resolving mediators [18,19].

## 3. The Influence of Triglycerides Reduction on CV Outcome

The Reykjavik and the European Prospective Investigation of Cancer (EPIC)-Norfolk studies showed that triglyceride levels had a similar effect as blood pressure and total cholesterol level [3]. However, after adjustment for established risk factors, this effect significantly diminished [3]. The meta-analysis of 29 studies that included 262,525 participants and 10,158 patients with coronary heart disease demonstrated similar results [3]. 

A meta-analysis that involved 197,270 participants who were treated with non-statin therapy with 25,218 major vascular events reported that triglyceride reduction was related to a decreased risk of major vascular events, even after adjustment for LDL-C lowering [20]. It seems that the benefits of marine-derived omega-3 fatty acids, especially high-dose eicosapentaenoic acid, exceed their lipid-lowering effects.

Interestingly, a recent study revealed an independent and inverse association between triglyceride levels and risk of all-cause mortality and cardiovascular mortality in patients with confirmed coronary artery disease (CAD) [4]. A subgroup analysis found similar results in patients with acute coronary syndrome and acute myocardial infarction [4]. The authors suggested the existence of the triglyceride paradox in patients with CAD [4]. Another investigation reported that low LDL cholesterol and low triglycerides were associated with significantly higher 30-day in-hospital mortality in patients with acute myocardial infarction [5]. The main contra arguments for the triglyceride paradox are obesity, lower social-economic status in these patients, the effect of heparin on triglyceride levels in acute coronary syndrome, and many confounding factors that could influence this association, but cannot be determined. 

## 4. The Effects of Fatty Acids

Omega-3 fatty acids reduce plasma triglyceride levels by lowering VLDL production, increasing VLDL clearance, or stimulating lipoprotein lipase activity. Eicosapentaenoic acid (EPA) reduced triglyceride levels without raising LDL levels, compared with docosahexaenoic acid (DHA), which increased LDL levels even when used at similar doses in patients with hypertriglyceridemia [21]. EPA and DHA can decrease apolipoprotein C-III level [22]. A reduction in apolipoprotein C-III induced a decrease in triglycerides through lipoprotein lipase activation [22]. Importantly, EPA and DHA induced an increase in HDL [23].

In addition to the hypotriglyceridemic effect, omega-3 fatty acids are related to anti-inflammatory, antithrombotic and antioxidant effects, as well as antiarrhythmogenic effects, the improvement of endothelial function, and insulin resistance.

DHA reduced secretion of interleukin (IL)-6 and IL-8 [24], and EPA decreased high-sensitivity C-reactive protein (hsCRP) in patients with hypertriglyceridemia compared to DHA [25]. Even though investigations showed that the efficacy of lowering hsCRP was similar in EPA and DHA [26], another study reported that the combination of EPA and DHA did not reduce hsCRP in patients with hypertriglyceridemia [27]. 

Omega-3 fatty acids significantly improved the serum total antioxidant capacity, increased glutathione peroxidase activity, and reduced malondialdehyde [28]. EPA inhibits the oxidation of apolipoprotein B-containing particles (such as LDL, VLDL, and small dense LDL) unlike DHA [29].

Omega-3 fatty acids affect platelet aggregation and have an antithrombotic effect through several mechanisms, such as generation of thromboxane A3 and prostacyclin, that have the cardioprotective and antithrombotic effect in patients treated with EPA and DHA. Interestingly, EPA supplementation may be more beneficial for males, while DHA may have more of an effect on platelet aggregation in females [30]. This suggests the potential influence of sex hormones and omega-3 fatty acids on reduced platelet aggregation in healthy subjects [30]. Some studies reported a more intensive antithrombotic effect of highly purified DHA than EPA [31]. The beneficial effect of omega-3 fatty acids in patients with coronary artery disease was recently proved in a study that involved only patients after acute myocardial infarction, demonstrating that patients with higher baseline values of omega-3 fatty acids had significantly lower risk of adverse clinical events [32]. 

## 5. Data from the Largest Clinical Trials

One of the first large clinical trials about the effects of omega-3 fatty acids—Japan EPA lipid intervention study (JELIS)—showed that eicosapentaenoic acid significantly reduced major coronary events in patients with a history of CAD and hypercholesterolemia, whereas there was no effect in patients without a history of CAD [33] (Table 1). These findings underlined the importance of omega-3 fatty acids in secondary prevention, but not in primary prevention. The authors primarily included patients with hypercholesterolemia who were treated with statins, which should emphasize the effect of omega-3 fatty acids on hypertriglyceridemia. This study was the first clinically randomized trial that provided some promising results in the treatment of hypertriglyceridemia with omega-3 fatty acids in secondary CV prevention.

The next large Outcome Reduction with an Initial Glargine Intervention (ORIGIN) trial that involved patients who were at high risk of CV events with impaired fasting glucose, glucose intolerance, or diabetes demonstrated that daily supplementation with 1g of n-3 fatty acids did not decrease CV risk [34] and confirmed the results of the JELIS study about the lack of positive effect in primary prevention [33]. Considering the fact that this study included a specific population with problems with glucose regulation and investigated the effect of insulin glargine, in addition to n-3 fatty acids, it would be justified to argue the effect of n-3 fatty acids was masked by the hypoglycemic effect of insulin [34]. A Study of Cardiovascular Events in Diabetes (ASCEND) included a similar, but not the same, population as the ORIGIN trial—diabetic patients also failed to show a significant reduction in risk of serious vascular events (non-fatal myocardial infarction or stroke, transient ischemic attack, or vascular death) between patients who received n-3 fatty acids and those who received a placebo [35]. The VITamin D and OmegA-3 TriaL (VITAL) investigated the effect of vitamin D and omega-3 fatty acids in the primary prevention of CVD and cancer among men 50 years of age or older and women 55 years of age or older, and, again, did not show any significant reduction in incidence of major CV events (myocardial infarction, stroke, or death from cardiovascular causes) in patients treated with omega-3 fatty acids in comparison with placebo [36]. These large trials significantly reduced the expectations of medical professionals from omega-3 fatty acids and their positive effect on CV outcome and particularly in primary prevention.

Reduction of Cardiovascular Events with Icosapent Ethyl–Intervention Trial (REDUCE-IT) significantly changed our perception about omega-3 fatty acids [6] (Table 1). This study involved patients with established CVD or diabetes and other risk factors who were treated with statins and who had increased triglyceride levels and well-controlled LDL levels [6]. Approximately 71% of all included patients already had CVD. The results of this study showed that the risk of ischemic events (non-fatal myocardial infarction, non-fatal stroke, coronary revascularization, unstable angina, or CV death) was significantly lower in patients with elevated triglyceride levels, despite the use of statins in those who received 2 g of icosapent ethyl twice daily than in those who received a placebo [6]. One should note that the dosage of omega-3 fatty acids was twice as high as in the other studies. Furthermore, some safety concerns were raised after this trial due to an increased number of hospitalizations due to atrial fibrillation and a trend of increased prevalence of serious bleeding events in patients treated with icosapent ethyl [6]. Subanalysis revealed that icosapent ethyl substantially decreased the risk of the first, subsequent, and total ischemic events (non-fatal myocardial infarction, non-fatal stroke, coronary revascularization, and unstable angina) [37]. The recent cost-effectiveness study reported that icosapent ethyl should be prioritized in patients with an established CVD—secondary prevention [38] (Table 1).

A Long-Term Outcomes Study to Assess STatin Residual Risk Reduction with EpaNova in HiGh Cardiovascular Risk PatienTs with Hypertriglyceridemia (STRENGTH) study compared omega-3 fatty acids (eicosapentaenoic acid and docosahexaenoic acid) with corn oil in statin-treated patients with high CV risk, hypertriglyceridemia, and low levels of HDL [39]. The authors found that the addition of omega-3 fatty acids in statin-treated patients at high CV risk did not decrease a composite outcome of major adverse cardiovascular events (cardiovascular death, non-fatal myocardial infarction, non-fatal stroke, coronary revascularization, or unstable angina requiring hospitalization) in comparison to patients treated with corn oil [39]. All patients were treated with the usual background therapies including statins [39]. Interestingly, a greater rate of gastrointestinal adverse events was observed in the omega-3 fatty acids group (24.7%) compared with corn oil-treated patients (14.7%) [39] (Table 1).

Clinical data from randomized clinical trials did not provide very encouraging results about the use of omega-3 fatty acids. On the other hand, pooled data in meta-analyses still support the use of this supplementation in patients with known CVD. These conflicting results need clarification and therefore much was expected from the EVAPORATE trial (Effect of Vascepa on Improving Coronary Atherosclerosis in People with High Triglycerides Taking Statin Therapy), which brought a completely new perspective on sagas about effects of omega-3 fatty acids [7,8].

### The EVAPORATE Trial: Happy Ending or Never-Ending Story

This trial was based on the REDUCE-IT study, but it included a small number of participants who underwent sophisticated coronary computed tomographic angiography in order to evaluate the volume and composition of coronary plaque in patients treated with icosapent ethyl [7,8]. All included patients had at least one angiographic stenosis (≥20% narrowing) and were treated with statins with a stable level of LDL (40 and 115 mg/dL) and hypertriglyceridemia (135–499 mg/dL). Coronary computed tomographic angiography was performed at baseline and after 9 and 18 months (Table 1). 

The results after the first follow-up did not show any significant change in low-attenuation plaque volume between the icosapent ethyl-group and placebo group [7]. Nevertheless, even after adjustment by baseline plaque, age, sex, diabetes, baseline triglyceride levels, and statin use, there was a significant slowing in the progression of total non-calcified, fibrous and calcified plaques [7]. After a complete follow-up of 18 months, the difference in plaque composition was even more notable. The volume of low-attenuation, fibro-fatty, and fibrous plaques was significantly reduced in the subjects who received icosapent ethyl (−17%, −34%, and −20%, respectively), while the volume of these plaques increased in the placebo group (+109%, +32%, and +1%, respectively) [8]. 

Another study that included 130 patients with acute coronary syndrome treated with high-dose statin therapy reported that eicosapentaenoic acid or eicosapentaenoic acid + docosahexaenoic acid therapy, in addition to high-dose statin therapy, did not significantly increase fibrous cap thickness in non-culprit plaques compared with high-dose statin therapy alone, but significantly increased fibrous cap thickness in patients with thinner fibrous caps [40]. This might provide a greater stabilizing effect on coronary plaques with vulnerable features. However, during the follow-up period of 12 months, there were no significant differences in major CV events (all-cause death, cardiac death, non-fatal myocardial infarction, stroke, coronary revascularization, or target lesion revascularization) between the control group and the other two groups [40].

It has already been well-documented that statins significantly slow the progression of atherosclerotic plaques [41,42]. The PARADIGM trial demonstrated that statins were associated with a slower progression of coronary atherosclerosis volume and a reduction in high-risk plaque features [41]. Statins did not change the severity of coronary artery stenosis but changed its phenotype [41]. The plaque composition in the statin group was significantly different from the control group. Patients who were treated with statins showed a faster progression of calcified plaques, but a slower progression of non-calcified plaques (fibrous and fibro-fatty plaques) than those in the control group [41,42]. 

Data coming from the EVAPORATE trial seem to be somewhat surprising because the effect of icosapent ethyl was stronger than with intensive statin therapy. The EVAPORATE study showed that the volume low-attenuation and fibro-fatty plaques in the placebo group increased by +109% and +32%, respectively [8]. This extraordinary increase in plaque volume in this short period of time (18 months) is difficult to explain from a medical point of view, and is rather the result of a small sample size, relatively low threshold value for triglyceride levels (135 mg/dL; 1.53 mmol/L) and definition of coronary stenosis (≥20%), and used statistical transformation. The intensity of statin therapy in the EVAPORATE trial is unknown, which also might impact the final results regarding plaque volume and composition. The REDUCE-IT study raised a question regarding if a placebo could be harmful by itself and might induce exacerbation of atherosclerosis during follow-up. This would result in false positive results of omega-3 fatty acids and their antiatherosclerotic potential in both studies (REDUCE-IT and EVAPORATE). This is not very likely, but it should be considered if a large discrepancy between active and placebo is noticed. Additionally, the effect of sample size in the EVAPORATE trial might be essential in obtaining positive results, because small trials are more likely to report larger beneficial effects than large trials, which could be partially explained by the lower methodological quality in small trials [43].

## 6. Controversies

The largest clinical trials about the effect of omega-3 fatty acids seem to be consistent in regard to inclusion criteria and used supplementation (type of omega-3 fatty acid), but they are actually very different. Some studies used only icosapent ethyl, the ester of eicosapentaenoic acid, or eicosapentaenoic acid itself [33,34,37], whereas others used a combination of eicosapentaenoic and docosahexaenoic acids [35,36,39]. Moreover, the dosage of omega-3 fatty acid was very different between trials. Only the latest trials, STRENGTH and REDUCE-IT, used a very high dosage of omega-3 fatty acids (4g) [37,39], whereas other studies used significantly lower dosages [35,36,37]. The placebo also varied between studies, from olive and mineral oil to corn and soybean oils, which could potentially be a limitation for appropriate comparison between different trials.

The inclusion criteria also significantly varied between trials. Some studies were focused on primary prevention [34,35,36], whereas others investigated primary and secondary prevention and patients with established CVD [33,37,39]. However, in some investigations, diabetes and dysregulation of glucose metabolism had a central role for study recruitment [34,35]. Additionally, basal therapy with statins significantly varied between studies, and it is not clear in the final results if treatment with statins could be completely adjusted for this very important concomitant therapy. One should notice that different populations and races were involved in different trials, which might significantly influence the final results. The effect of omega-3 fatty acids is not the same in East Asian populations as in Western populations. The dosage of omega-3 fatty acid in different populations should probably not be the same.

Omega-3 fatty acids were introduced as a supplemental therapy in the treatment of dyslipidemias, and particularly hypertriglyceridemia at the very beginning. However, studies showed that their influence on triglyceride levels is modest in comparison with effect of statins on LDL levels, which means that omega-3 fatty acids have some additional positive CV effects beyond triglycerides reduction. The REDUCE-IT study reported a significant triglycerides reduction (18.6%) in the icosapent ethyl group, but also a reduction in HDL and increase in LDL level in the same group [6]. The relatively small differences in LDL levels between the groups in the REDUCE-IT trial would not likely explain the 25% lower risk observed with icosapent ethyl, and a post hoc analysis reported a similar lower risk, irrespective of whether there was an increase in LDL among the patients in the placebo group [6].

Antihypertensive effect [28], antithrombotic effect [28,29], and the reduction in oxidative stress [44] seem reasonable mechanisms that could explain the potential positive effect of omega-3 fatty acids. However, the increase in LDL and decrease in HDL levels are raising concerns about the use of omega-3 fatty acids and their effects on the lipidemic profile.

The follow-up period and primary endpoints were similar in different trials [33,34,35,36,37,38,39]. However, the results varied from positive effects for secondary prevention in the JELIS and REDUCE-IT trials to neutral effects in the rest of the trials. It seems that the only agreement between all trials is a lack of evidence for the beneficial effect of omega-3 fatty acids in primary CV prevention.

## 7. Current Perspective

The REDUCE-IT trial significantly changed the perspective on omega-3 fatty acids and their use for the therapy of hypertriglyceridemia. In December 2019, the US Food and Drug Administration gave approval to Vascepa (icosapent ethyl) in the treatment of hypertriglyceridemia in order to reduce CV risk in high-risk patients. Following these recommendations, the European Medicines Agency’s (AMA) Committee for Medicinal Products for Human Use has recommended icosapent ethyl for the prevention of CV in high-risk patients. Secondary prevention is the focus of recommendations in both US and Europe. 

The appropriate dosage of eicosapentaenoic acid might be 1.8 g/day for East Asians, such as Korean, Japanese, and Chinese, and icosapent ethyl 4 g/d for Western nations, because a plasma eicosapentaenoic acid level (170 μg/mL in a Japanese population) after 1.8 g/day treatment was equivalent to the level that was obtained after 4 g/day of icosapent ethyl in a Western population (183 μg/mL) [45,46].

## 8. Experts’ Opinions

It is clear that many questions need to be answered in the near future, particularly due to the fact that icosapent ethyl is approved by the FDA and EMA for patients with hypertriglyceridemia in secondary CV prevention, as well as in patients with hypertriglyceridemia and diabetes with additional CV risk factor(s). The latest findings from the STRENGTH trial that has just been published confirmed no association between use of high-dose EPA and DHA and reduction in major CV events [18], which is why investigators decided to finish the trial earlier than expected. The authors did not find that EPA to be beneficial or DHA harmful among patients treated with fish oil [46]. The argument from the REDUCE-IT and EVAPORATE trials [6,7] is that they used a different active component—icosapent ethyl—that increased EPA significantly, without raising DHA, which occurred in the STRENGTH trial. Findings from the large study are inclined toward risk reduction in secondary prevention, even though it is not clear which mechanism is responsible for this reduction. It seems that anti-inflammatory mechanisms, and potentially some additional mechanisms, are more important for CV risk reduction than a simple decrease in triglycerides because other medications, such as fibrates, reduce triglyceride levels even more than omega-3 fatty acids, but they were not proved to reduce CV risk. Nevertheless, we should also be aware of reported side effects, particularly atrial fibrillation and hemorrhage. Therefore, prescribing carefully is advisable at the beginning of commercial and wide usage. We believe that post-marketing research will provide reliable data from clinical practice that will finally give answers on all aforementioned questions.

## 9. Conclusions

Data regarding omega-3 fatty acids and CV protection are conflicting, but the latest studies provided some promising results and added them in a small armamentarium of medications against hypertriglyceridemia. Moreover, omega-3 fatty acids, in large doses, are not considered as supplements any longer but as drugs. There are many questions that need to be answered in the future, particularly the effectiveness of omega-3 fatty acids in primary prevention, as well as the independent effect of statins, appropriate dosage in different populations, and safety profile, particularly the risk of atrial fibrillation and hemorrhage. It seems that there is still a long way to go for the full acceptance of omega-3 fatty acids in everyday clinical practice. Nevertheless, we should strongly consider this kind of therapy in statin-treated patients with hypertriglyceridemia who are at high- and very high CV risk and gain more experience and information about this drug from post-marketing studies and future clinical trials.

## Figures and Tables

**Table 1:** The overview of the largest clinical trials about omega-3 fatty acids and cardiovascular outcome.

Reference	Sample Size	Omega-3 Fatty Acid	Inclusion Criteria	Follow-Up Period (Years)	Main Findings
JELIS [33]	18,645	EPA	Patients with or without CAD (previous MI, PCI or confirmed angina pectoris)	4.6	EPA reduced all-cause mortality in secondary, but not in primary prevention.
ORIGIN [34]	12,536	n-3 fatty acids	Patients at high risk of CV events and with imapired fasting glucose, glucose intolerance or DM	6.2	No reduction in rate of CV events (non-fatal MI or stroke, death from CV cause or arrhythmia) among patients who received omega-3 fatty acids in patients at high CV risk in primary prevention.
ASCEND [35]	15,480	EPA + DHA	Patients older than 40 years with DM, but without CVD	7.4	No effect on CV events (non-fatal MI or stroke, transient ischemic attack, or vascular death, excluding confirmed intracranial hemorrhage) among patients who received omega-3 fatty acids.
VITAL [36]	25,871	EPA + DHA	Healthy subjects (men > 50 years and women > 55 years)	5.3	No effect on CV events (MI, stroke, CV death) among patients who received omega-3 fatty acids.
REDUCE-IT [6]	8,179	Icosapent ethyl	Patients with established CVD or DM on statin therapy with increased TG	4.9	The risk of ishemic events (CV death, MI, revascularization, unstable angina) was significantly lower in patients tretaed with icosapent ethyl.
EVAPORATE [7]	80	Icosapent ethyl	Patients with confirmed coronary artery stenosis, on statin therapy with elevated TG	1.5	Patients on icosapent ethyl therapy experienced a significant reduction in low-attenuation plaque volume and thickening of fibrotic cap, which stabilizes plaque and prevents its rupture.
STRENGTH [37]	13,078	EPA + DHA	Established CVD or high risk for CVD	3.5	No significant effect of omega-3 fatty acids on CV death, non-fatal MI and stroke, coronary revascularization, and unstable angina.

CAD—coronary artery disease, CV—cardiovascular, CVD—cardiovascular disease, DHA—docosahexaenoic acid, DM—diabetes mellitus, EPA—eicosapentaenoic acid, MI—myocardial infarction, PCI—percutaneous coronary intervention.
